# The prevalence of acute stress disorder after acute myocardial infarction and its psychosocial risk factors among young and middle-aged patients

**DOI:** 10.1038/s41598-022-11855-9

**Published:** 2022-05-10

**Authors:** Minjuan Wu, Wenqin Wang, Xingwei Zhang, Junhua Li

**Affiliations:** 1 Hangzhou Normal University, Hangzhou, 311121 Zhejiang China; 2 The Affiliated Hospital of Hangzhou Normal University, Hangzhou, 310015 Zhejiang China

**Keywords:** Psychology, Cardiology

## Abstract

Young and middle-aged people are vulnerable to developing acute stress disorder (ASD) following acute myocardial infarction (AMI). This study aims to explore the factors that contribute to ASD in young and middle-aged AMI patients. 190 AMI patients aged 18 to 60 years were enrolled in this study. We assessed the association between ASD and demographic data, adult attachment, and social support. This study examined a total of 190 young and middle-aged people. Among them, 65 participants were diagnosed with ASD, representing a 34.21% positive rate. Multivariate stepwise regression showed that adult attachment, infarct-related artery, social support, in-hospital complications are the main factors affecting ASD. Path analysis showed that social support had mediated the relationship between adult attachment and ASD. The incidence of ASD in young and middle-aged patients with AMI is high. Social support plays an important role in adult attachment and ASD relationships. Adult attachment and social support should be incorporated into post-traumatic cardiac rehabilitation to help patients cope with traumatic occurrences.

## Introduction

Acute myocardial infarction (AMI) is a severe type of coronary heart disease. As a traumatic event, AMI condition can cause acute stress disorders (ASD) like anxiety, depression, numbness, and stress response, leading to increased sympathetic excitability. High sympathetic excitability leads to associated pathophysiological changes, gradually promoting or aggravating myocardial infarction and heart failure. Most people's ASD symptoms may improve after a few weeks or months. However, some people cannot recover and repeatedly suffer from numbness, avoidance, intrusion, and other symptoms, eventually developing posttraumatic stress disorder (PTSD).

PTSD is a severe mental health problem that has received considerable attention, while ASD in the early stage of trauma is often overlooked. ASD is defined as the emotional, physical, and dissociative reaction during a traumatic event and lasts for less than one month^1^. People with ASD usually exhibit behaviors such as crying and apathy towards life^2^. In addition to affecting people's psychological state, ASD can also cause physiological changes such as pain and decreased immune resistance, impairing one’s quality of life^3^.

ASD was found to be prevalent in 18% of patients with acute coronary syndromes (ACS), such as AMI^4^. ASD is associated with impaired quality of life and adverse cardiovascular consequences after ACS^5,6^. Age is a predictor of ASD, and Ghada et al. found that the risk of ASD in young persons following stressful events is greater than in the elderly^7^. Being the center of the social labor force, the young and middle-aged people are at a point in their life where professional development is critical. The disease’s impact on their lives and the economy is thus significantly greater than that of other age groups. However, the symptoms of ASD following AMI and the factors influencing individual susceptibility in young and middle-aged people remain unclear. Therefore, additional research on the psychological stress response of young and middle-aged AMI patients is necessary.

Additionally, it was found that an individual's social support environment has a significant impact on their psychological distress. Social support refers to social connections with other individuals, groups, and the larger community^8^. According to Norris's social support deterioration deterrence model, social support acts as a protective "cushion" in stress response. Individuals who receive social support are less likely to be impacted by stressful events^9,10^. Therefore, AMI may aggravate the severity of ASD symptoms by jeopardizing young and middle-aged people’s social support systems.

Adult attachment patterns are the basis of human relationships, influencing the association between social support and psychological distress^11^. As a traumatic event, AMI can trigger the patient's attachment system. Attachment is defined as an intimate and lasting emotional connection between individuals and others. It plays an essential role in cognition, emotion, and social behavior^12^. The effect of attachment on ASD in young and middle-aged AMI patients has not been explained in current related studies. As such, this study intends to explore the current situation of ASD in young and middle-aged AMI patients. This study aims to establish the severity of ASD and predictors of psychological distress among AMI patients more precisely.

## Methods

### Participants and procedure

**Figure 1:**
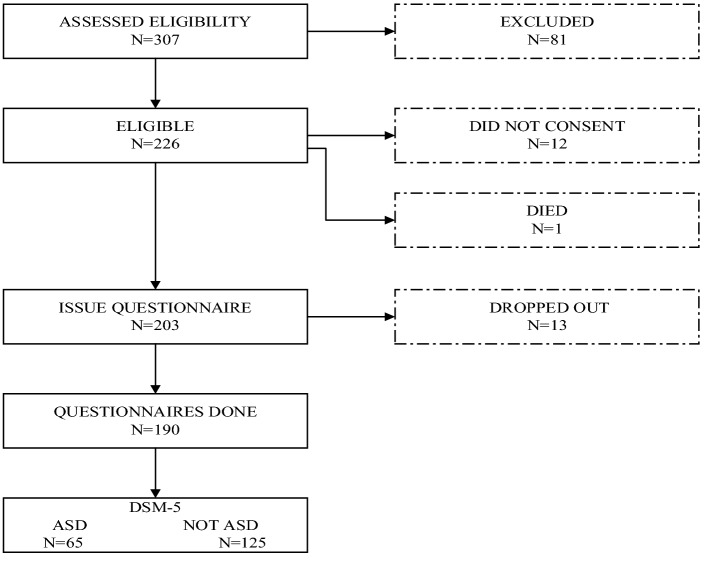
Patient flow chart.

The subjects were recruited between January 2019 and December 2020 at The Affiliated Hospital of Hangzhou Normal University. Patients meeting the following criteria were eligible for the study. Inclusion criteria: (1) diagnosed with AMI; (2) aged between 18 and 60 years old. Exclusion criteria: (1) combined with previous complications; (2) diagnosed with dementia or other psychiatric diseases; (3) having hearing or communication impairment; (4) experienced traumatic events within half a year. The subjects were assessed for the presence of ASD through a structured interview based on the DSM-5 (the 5th edition of the Diagnostic and Statistical Manual of Mental Disorders) criteria. The diagnostic criteria for ASD according to DSM-5 mainly include: (1) witnessed, learned, or underwent an event(s) involving death, actual or threatened serious injury, actual or threatened physical or sexual violation; (2) exhibited an array of clinically significant posttraumatic re-experience, dissociative, avoidance and/ or arousal symptoms^13^. The study flow chart is illustrated in Fig. 1.

A power analysis was conducted using the G*Power 3.1 software to calculate the minimum sample size required to achieve sufficient power for the statistical analyses involved. A sample size of 152 was estimated to be required, with a power level of 0.95 and an alpha of 0.05. A total of 203 questionnaires were issued in this study, with 190 completed questionnaires returned, resulting in an effective rate of 93.6%. The subjects’ mean age was 49.99 (± 8.07) years -ranging from 23 to 60 years. Most of the subjects were male (92.11%), and their mean age was 49.69 (± 8.09) years, while female subjects accounted for 7.89%, with their mean age being 51.93 (± 7.89) years.

### Research ethics

This study was approved by the Ethics Committee of The Affiliated Hospital of Hangzhou Normal University (IRB's registration number: 2019 Ethics 02-HS-46). This study complies with the international declaration of Helsinki, the ethical examination and approval measures for biomedical research involving human subjects, and applicable laws and regulations. Each participant was given an information sheet (mainly about demographic characteristics and questionnaires used in this study) and a consent form prior to their participation in the study. Informed consent from each participant was obtained before the study. Printed questionnaires were distributed to those who agreed and consented to participate. All participants were assured that their refusal or withdrawal from the study would not affect their treatment course.

### Measures

#### Demographics

Demographic characteristics include sex, marital status, education, occupation, payment, number of stent implantation, in-hospital complications, cardiac function (Killip class at admission), infarct-related artery, AMI-related knowledge, smoking, alcohol consumption, substance abuse.

‘Payment’ was classified into three categories: "rural medical insurance" where the patient is required to pay 50% of their medical expenses; "urban medical insurance" where the patient is required to pay 30% of their medical expenses and "self-paying" where patients need to pay all their medical expenses by themselves.

‘In-hospital complications’ include in-hospital hemorrhagic stroke, ischemic stroke, cardiopulmonary resuscitation, heart failure, hypotension requiring vasopressors and arrhythmia.

‘AMI-related knowledge’ was identified by evaluating the patients’ knowledge on the infarct-related artery, laboratory examination results, treatment measures, and possible complications of AMI (4 items). Their knowledge was classified into three levels: "completely unaware" where the patient was unaware of all 4 items; "partly aware" where the patient knows at least one item and "fully aware" where the patient fully understands all 4 items.

#### Stanford Acute Stress Reaction Questionnaire (SASRQ)

The 30-item Stanford Acute Stress Reaction Questionnaire (SASRQ) measured participants’ ASD^14^. The questionnaire assesses dissociation (10 items mainly evaluating patients’ cognitive changes such as memory loss, their decline in environmental clarity and emotional changes such as numbness and lack of emotional response); re-experience of trauma (6 items primarily evaluating patients’ physiological reactions like physical symptoms caused by traumatic events and behavioral changes such as constantly having unnecessary forced thoughts of past traumatic events.); avoidance (6 items mainly assessing behavioral changes in patients such as being away from others and avoiding things associated to their traumatic experiences); anxiety and hyperarousal (6 items mainly assessing patients’ behavioral changes such as sleep changes and panic attacks, cognitive changes like decreased attention and emotional changes such as tension, anxiety, and irritability); and functional impairment (2 items mainly evaluating patients’ physiological reactions such as impairment of physical function). SASRQ is scored on a 5-point Likert scale ranging from 0 (not experienced) to 5 (very often experienced). The score range is 0–150 points. A total score of SASRQ ≥ 40 is positive for acute stress disorder. The higher the score, the more severe the patient’s acute stress disorder. The Cronbach's α coefficient of the scale was 0.87–0.95.

#### Experiences in Close Relationships Inventory (ECR)

The adult attachment was assessed using the Experiences in Close Relationships Inventory (ECR)^15^. ECR produces two scores: attachment-related avoidance and attachment-related anxiety. The scale contains 36 questions adopting the 7-level scoring method: strongly disagree, disagree, somewhat disagree, not sure, somewhat agree, agree and strongly agree, which are recorded as 1–7 points, respectively. Questions 3, 15, 19, 22, 25, 27, 29, 31, 33, and 35 are scored reversedly. The sum score of odd number questions equals the score for attachment-related avoidance, while the total score of even number questions refers to the attachment-related anxiety score. The higher the score, the higher the degree of attachment-related anxiety or avoidance. The Cronbach's α coefficient of the scale was 0.79–0.82.

#### Social Support Rating Scale (SSRS)

Patients’ social support was measured using Xiao's Social Support Rating Scale (SSRS)^16^. The scale has ten items, including objective support (3 items), perceived support (4 items), and support utilization (3 items). The score range is 12–66 points. A score below 35 points indicates a low level of social support, 35–45 points indicates a moderate level of social support, and a score greater than 45 indicates a high level of social support. The scale demonstrated impressive validity and reliability for the Chinese population (Cronbach's α = 0.949)^17^.

### Statistical analysis

Demographic characteristics, ASD, social support and adult attachment, are all described using observed values, percentages, quartile, means, and standard deviations. The differences in the participants’ ASD based on demographic characteristics were analyzed using the nonparametric rank-sum test. The relationship between social support, adult attachment, and ASD was assessed using Spearman’s correlation coefficients. Additionally, multiple linear regression was used to determine the factors that influence individuals' ASD. The dependent variable was set as ASD, and the independent variables were set as perceived support, in-hospital complications, attachment-related avoidance, and attachment-related anxiety. Path analysis was performed to evaluate the mediating effect of social support on the relationship between adult attachment and ASD. The fit indices used included the root mean square error of approximation (RMSEA), comparative fit index (CFI), and normed fit index (NFI). SPSS20.0 and AMOS17.0 were used for all analyses, and the statistical significance was set at P < 0.05 (2-tailed).

## Results

### Preliminary analysis

A total of 190 young and middle-aged people were investigated in this study. Among them, 65 were diagnosed with ASD, with a positive rate of 34.21%. Since the total score and each dimension of ASD do not conform to the normal distribution, it was described by median (M) and interquartile spacing (P25, P75) (see Table 1). The results showed that the main symptoms of ASD were hyperarousal, reexperience and dissociation. Table 2 shows the differences in the participants’ ASD based on their demographic characteristics. The results showed that ASD was significantly correlated with in-hospital complications (Z = − 2.639, *p* = 0.008), infarct-related artery (H = 25.840, *p* < 0.001), and AMI-related knowledge (H = 7.949, *p* = 0.019).

**Table 1:** Score of acute stress disorder in young and middle-aged patients with acute myocardial infarction (n = 190).

Variables	Items	Points [M(P25, P75)]
SASRQ total	30	35.00 (25.00,42.00)
Dissociation	10	8.00 (4.00,15.00)
Reexperience	6	7.00 (3.00,10.00)
Hyperarousal	6	12.00 (8.00,18.00)
Avoidance	6	2.00 (0.00,7.00)
Function impairment	2	5.00 (4.00,5.00)

SASRQ: Stanford Acute Stress Reaction Questionnaire.

**Table 2:** Differences in the participants’ ASD based on demographic characteristics [n = 190, M(P25, P75)].

Variables	Subgroups	n	Total SASRQ	Statistical value	*p*
Sex	Male	175	35.00 [25.00,42.00]	Z = − 0.491	0.623
	Female	15	35.00 [33.00,49.00]		
Marital status	Married	178	35.00 [20.75,75.00]	H = 2.985	0.611
	Single	8	52.00 [28.75,48.75]		
	Widowed/divorced	4	36.00 [29.25,109.50]		
Education	Primary school and below	58	39.00 [26.50,65.00]	H = 6.051	0.195
	Junior high school	73	36.00 [25.00,45.50]		
	High school	36	30.50 [25.00,37.00]		
	Junior college	12	27.50 [21.75,37.00]		
	Bachelor degree and above	11	34.00 [24.00,39.00]		
Profession	Farmer	37	36.00 [24.50,45.50]	H = 8.698	0.122
	Worker	94	35.00 [25.00,41.00]		
	Self-employed	17	41.00 [30.00,107.00]		
	Civil servant	2	22.50 [21.00,–]		
	Retired	13	37.00 [24.50,66.00]		
	Others	27	33.00 [25.000,42.00]		
Alcohol consumption	Never	68	37.00 [25.00,65.00]	H = 1.654	0.437
	Former	26	34.00 [24.00,45.00]		
	Current	96	35.00 [25.00,41.00]		
Smoking	Never	101	35.00 [25.00,47.25]	H = 2.299	0.513
	Former	43	35.00 [25.00,41.00]		
	Current < 20 cigarettes	29	34.00 [24.50,39.00]		
	Current ≥ 20 cigarettes	17	39.00 [26.00,73.00]		
Substance abuse	Yes	5	34.00 [21.00,51.00]	Z = − 0.635	0.525
	No	185	35.00 [25.00,42.00]		
Payment	Self-paying	13	36.00 [27.00,62.75]	H = 2.930	0.234
	Rural medical insurance	80	35.00 [21.50,41.00]		
	Urban medical insurance	97	35.50 [24.50,42.00]		
Number of stent implantation	1	123	36.00 [25.00,42.00]	H = 3.835	0.280
	2	53	35.00 [23.00,41.50]		
	3	10	39.00 [23.75,65.50]		
	≥ 3	4	26.50 [21.25,34.00]		
In-hospital complications	No	159	35.00 [24.00,41.00]	Z = − 2.639	**0.008**
	Yes	31	41.00 [33.00,73.00]		
Killip class at admission	Class I	168	35.00 [25.00,42.00]	H = 1.242	0.741
	Class II	12	36.00 [28.00,52.25]		
	Class III	4	38.50 [30.00,60.50]		
	Class IV	6	31.50 [20.75,47.00]		
Infarct-related artery	Left main	16	41.00 [28.50,54.25]	H = 25.840	** < 0.001**
	Left anterior descending artery	135	34.00 [24.00,39.00]		
	Right coronary artery	27	56.00 [35.00,75.00]		
	Left circumflex artery	12	69.00 [37.50,78.75]		
AMI-related knowledge	Completely unaware	57	39.00 [25.00,67.00]	H = 7.949	**0.019**
	Partly aware	51	36.00 [25.00,42.00]		
	Fully aware	82	34.00 [24.00,37.50]		

H: Kruskal–Wallis H Test; Z: Mann–Whitney U Test.

Significant values are given in bold.

### Relationships between ASD, social support and adult attachment

The score of social support for this study was 36.01 (± 9.72) points: 8.83 (± 2.76) points for objective support, 20.23 (± 5.89) for perceived support and 6.94 (± 2.77) for support utilization. The score of attachment-related anxiety was 48.06 (± 14.83) points and that of attachment-related avoidance was 63.44 (± 13.57). The correlations between ASD, social support and adult attachment are shown in Table 3. ASD revealed a significant negative correlation with social support (ρ = − 0.334, *p* < 0.01), objective support (ρ = − 0.291, *p* < 0.01), perceived support (ρ = − 0.313, *p* < 0.01), and support utilization (ρ = − 0.251, *p* < 0.01). Additionally, ASD demonstrated a significant positive correlation with attachment-related avoidance (ρ = 0.374, *p* < 0.05) and attachment-related anxiety (ρ = 0.402, *p* < 0.05).

**Table 3:** Correlations between ASD, social support and adult attachment (n = 190, ρ).

Variables	Dissociation	Reexperience	Hyperarousal	Avoidance	Function impairment	ASD
Social support	− 0.237**	− 0.212**	− 0.244**	− 0.317**	− 0.227**	− 0.334**
Objective support	− 0.274**	− 0.156*	− 0.143*	− 0.269**	− 0.126	− 0.291**
Perceived support	− 0.196**	− 0.166*	− 0.256**	− 0.297**	− 0.214**	− 0.313**
Support utilization	− 0.197**	− 0.236**	− 0.194*	− 0.249**	− 0.219**	− 0.251**
Attachment-related Avoidance	0.311**	0.256**	0.249**	0.289**	0.175*	0.374**
Attachment-related anxiety	0.201**	0.386**	0.341**	0.223**	0.178*	0.402**

**p < 0.01; *p < 0.05.

### Factors influencing ASD

Multivariate regression was used to find the components that were independently associated with ASD. The multiple linear regression analysis was conducted using the ASD total score as the dependent variable and the factors of statistical significance in nonparametric rank-sum test and correlation analyses as the independent variables. The dummy variables were set for categorical variables (the values of the independent variables are shown in Table 4, α_inclusion_ = 0.05, α_exclusion_ = 0.10). The results revealed a significant regression model (F[9,180] = 11.404, *p* < 0.001), with an adjusted coefficient of determination (adjusted R^2^) of 0.331 for the power interpretation of the model. The contribution of independent variables to patients’ASD was sequenced as follows: attachment-related anxiety > infarct-related artery > perceived support > in-hospital complications > attachment-related avoidance based on the comparison of absolute values among variables’ standardized regression coefficients (see Table 5).

**Table 4:** Evaluation of independent variables.

Independent variables	Evaluation method
In-hospital complications	Yes = 0, no = 1
Infarct-related artery	The dummy variables were set with the baseline of "left main"
	Dummy variable X_1_ (left main = 0, left anterior descending artery = 1, right coronary artery = 0, left circumflex artery = 0)
	Dummy variable X_2_ (left main = 0, left anterior descending artery = 0, right coronary artery = 1, left circumflex artery = 0)
	Dummy variable X_3_ (left main = 0, left anterior descending artery = 0, Right coronary artery = 0, left circumflex artery = 1)
AMI-related knowledge	The dummy variables were set with the baseline of "completely unaware"
	Dummy variable X_4_ (completely unaware = 0, partly aware = 1, fully aware = 0)
	Dummy variable X_5_ (completely unaware = 0, partly aware = 0, fully aware = 1)
Objective support	Numerical variable
Perceived support	Numerical variable
Support utilization	Numerical variable
Attachment-related avoidance	Numerical variable
Attachment-related anxiety	Numerical variable

**Table 5:** Multivariate stepwise regression results of ASD (n = 190).

Variables	Regression coefficient	Standard error	Standardized regression coefficient	t	*p*
(Constant)	15.752	13.175	–	1.196	0.233
**Infarct-related artery (reference: "left main")**					
Left anterior descending artery	0.349	5.900	0.006	0.059	0.953
Right coronary artery	18.052	6.626	0.247	2.725	**0.007**
Left circumflex artery	12.659	8.043	0.121	1.574	0.117
**AMI-related knowledge (reference: "completely unaware"**					
Partly aware	− 3.135	4.230	− 0.055	− 0.741	0.460
Fully aware	− 3.746	4.093	− 0.073	− 0.915	0.361
Attachment-related anxiety	0.377	0.115	0.219	3.291	**0.001**
Attachment-related avoidance	0.294	0.133	0.156	2.212	**0.028**
Perceived support	− 0.668	0.299	− 0.154	− 2.230	**0.027**
In-hospital complications	9.600	4.313	0.139	2.226	**0.027**

Coefficient of determination: R^2^ = 0.363, adjusted R^2^ = 0.331, F = 11.404, *p* < 0.001.

Significant values are given in bold.

### The mediating effect of social support on the relationship between adult attachment and ASD

Hierarchical multiple regression analyses were conducted with perceived support and ASD as the dependent variables. Demographic characteristics were treated as the control variable, and adult attachment was entered as the independent variable. Table 6 presents the results of regression analyses. Attachment-related avoidance (β = 0.185, *p* < 0.01), attachment-related anxiety (β = 0.232, *p* < 0.01), and perceived support (β = − 0.193, *p* < 0.05) had significant effects on ASD.

**Table 6:** Hierarchical regression analysis for ASD.

Variables	Step 1(β)	Step 2(β)	Step 3(β)
Infarct-related artery	0.202*	− 0.058	0.191*
AMI-related knowledge	− 0.107	0.241**	− 0.060
In-hospital complications	0129	0.044	0.138*
Attachment-related avoidance	0.234**	− 0.249**	0.185**
Attachment-related anxiety	0.240**	− 0.043	0.232**
Perceived support	–	–	− 0.193
F	16.584**	8.175**	15.800**
R^2^	0.311	0.182	0.341

**p < 0.01; *p < 0.05.

**Figure 2:**
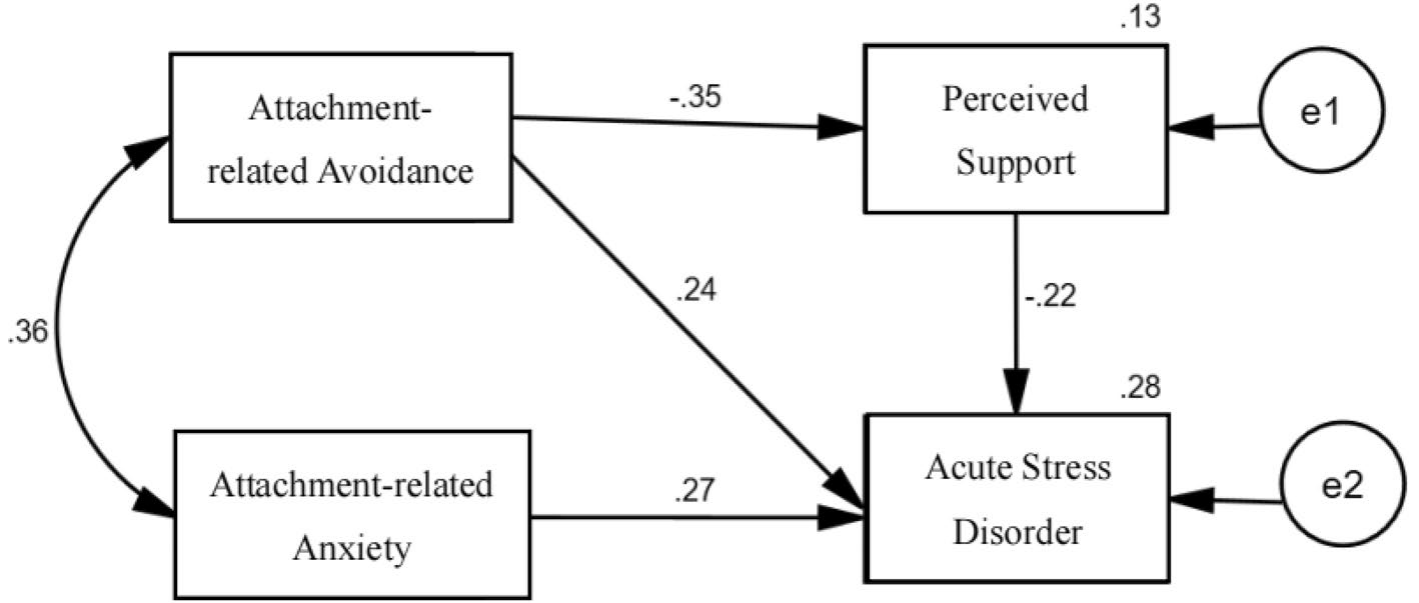
Path model explaining the effects of determinants.

Path analysis was used to construct an ASD prediction model based on perceived support and adult attachment. The fit indices indicated that the path model had a good fit to the data (χ^2^/df = 1.046, GFI = 0.997, CFI = 1, NFI = 0.991, TLI = 0.997, IFI = 1, RMSEA = 0.016). The results are shown in Fig. 2 and Table 7, and they revealed that perceived support had significant direct effects on ASD (β =  − 0.22, *p* < 0.05). The direct pathways from attachment-related avoidance to perceived support (β = − 0.35, *p* < 0.05) and ASD (β = 0.24, *p* < 0.05) were statistically significant. The bootstrapping results indicated that the indirect pathways between attachment-related avoidance and ASD through perceived support were significant (*p* < 0.05).

**Table 7:** Decomposition of standardized effects from the path model.

Effect	Path	Effect size	95%CI	
			Upper bounds	Lower bounds
Direct effects	Attachment-related avoidance → perceived support	− 0.354	− 0.237	− 0.461
	Attachment-related avoidance → ASD	0.235	0.361	0.102
Indirect effects	Attachment-related avoidance → perceived support → ASD	0.079	0.138	0.034
Total effects	Attachment-related avoidance → ASD	0.314	0.429	0.189
	Attachment-related anxiety → ASD	0.268	0.409	0.113
	Perceived support → ASD	− 0.224	− 0.095	− 0.341

## Discussion

This study examined the relationship between ASD and adult attachment and social support in young and middle-aged AMI patients, all while considering the potential impact of demographics. 34.21% of the participants developed ASD after percutaneous coronary intervention (PCI), which is higher than previously reported^4^. Roland found that AMI patients with ASD or PTSD were younger than those without, although their coronary heart disease severity was relatively mild^18^. This report confirmed that young and middle-aged people are more likely to develop ASD after experiencing cardiovascular events.

Our study showed that right coronary artery occlusion was associated with ASD. At present, there is no research report on the effect and mechanism of right coronary artery occlusion on ASD. We hypothesized that this could be due to different creatine kinase culmination. Sochman et al. reported that creatine kinase culmination (t-peak) is influenced by the necrosis site; for patients with infarction in the right coronary artery area, t-peak was 17.7 ± 4.7 h, while t-peak was 13.2 ± 4.6 h (*p* < 0.001)^19^ for those with infarction in the left ventricle. Creatine kinase culmination reveals a significant positive correlation with infarct severity^20^. Anxiety or PTSD is more frequently observed in people with higher disease severity^21,22^. Considering ASD is a subjective psychological measure index, future studies could explore the relationship between the infarct-related artery and objective psychological measure indexes such as epinephrine and dopamine.

Additionally, increased disease severity results in increased medical expenses and recovery time for patients. When the economy affects regular treatment and life, patients' perceived social support decreases^23^. Similar to previous studies, we found that social support helps deter negative emotions^24^. According to Norris et al.’s social support deterioration deterrence model, social support, as an external protective factor, plays an essential role in buffering the adverse effects of stress response^10^. Social support includes the visible and objective material or emotional support that individuals obtain from their social network relationships and the emotional experience of feeling respected, supported, and understood in society. Many studies have shown that perceived social support is more natural and effective for individuals and can better predict their mental health levels^25^. As a supportive resource, perceived social support can promote communication between participants and their families, alleviating their fear caused by AMI.

Notably, this study showed a positive correlation between attachment-related anxiety, attachment-related avoidance and ASD in AMI patients. Previous research showed that insecure attachment style (greater attachment-related avoidance or anxiety) predicted greater anxiety, depression, fasting blood glucose and glycosylated hemoglobin. Insecure attachment style is associated with poorer health outcomes in coronary heart disease patients experiencing traumatic stress^26^. As a traumatic stress, AMI can trigger the attachment system in patients; those with greater attachment-related anxiety are eager to get help from others but generally lack self-confidence and have abandonment issues. Therefore, they often exaggerate the stress events they encounter to attract attention from others, increasing their psychological pressure^12^.

On the physiological level, patients with greater attachment-related anxiety secrete more cortisol when faced with stressful events^27^. Previous studies have shown that excess cortisol may induce major depression disorder in individuals^28^. In addition, patients with greater attachment-related avoidance usually treat others with a negative attitude and believe that their interpersonal relationship is unreliable—they are unable to initiate engagement with others and may even avoid seeking help^29^. Girme et al. suggested that receiving low-to-moderate practical support from one’s partner increased distress risk in avoidant participants^30^. Therefore, young and middle-aged AMI patients with greater attachment-related avoidance will not actively seek help from medical staff and their families and avoid communication with others. This might strain their relationships and add additional barriers in processing their trauma effectively^31^.

### Relevance to clinical practice

In our study, social support was found to be related to young and middle-aged AMI patients’ psychological health. Healthcare institutions should offer psychological counseling to patients to relieve their stress. Medical staff should pay attention to their patients’ psychological conditions and not simply focus on physical problems. For example, each ward can be equipped with a psychologist to provide counseling to needy patients, which might help young and middle-aged AMI patients get some psychological relief. The medical staff could motivate and improve patients’ social support by using brochures or videos to encourage family members’ involvement in the treatment process. Additionally, the present study suggests that adult attachment may assist in identifying those at risk of developing psychological problems. Adult attachment can be assessed routinely to help predict psychological problems in patients. Given that ASD patients are predisposed to developing PTSD, follow-up sessions with young and middle-aged AMI patients are crucial.

## Limitations

This study has several limitations. Firstly, participants in this study were recruited using the convenient sampling method, so they may not be representative of all young and middle-aged AMI patients in China. Secondly, according to DSM-5, female patients are more prone to develop acute stress disorder. Previous research showed that women are more likely to develop ASD after traumatic experiences^32^. However, our study did not support any association between sex and ASD, although the total SASRQ score for female patients was slightly higher than that of male patients. This is consistent with Marie-Anne Roberge’s research results^33^ and suggests that it is necessary to collect more samples from different regions to explore ASD symptoms further and their relationship with sex in AMI patients. Thirdly, cross-sectional data analysis cannot be used to explain causality directly. Future longitudinal studies may be needed to confirm our findings.

## Conclusions

Despite the above-mentioned limitations, our study still demonstrates acute psychological reactions related to AMI and related factors that can reduce ASD symptoms (such as adult attachment and social support). Identifying the risk and protective factors in early AMI treatment is essential to prevent future ASD. Existing research reports also can provide reference significance. Since the AMI severity is related to ASD, the medical personnel can reduce negative emotions by continuously improving AMI’s first aid process and minimizing related in-hospital complications. In addition, medical staff should evaluate adult attachment and social support as soon as possible, adjust the nursing plan appropriately and encourage family members’ engagement in the treatment process to prevent ASD occurrence.

## References

[1] Bryant RA, Harvey AG, Dang ST, Sackville T, Basten C (1998). Treatment of acute stress disorder: A comparison of cognitive-behavioral therapy and supportive counseling. J. Consult. Clin. Psychol..

[2] Marin MF, Geoffrion S, Juster RP, Giguère CE, Marchand A, Lupien SJ, Guay S (2019). High cortisol awakening response in the aftermath of workplace violence exposure moderates the association between acute stress disorder symptoms and PTSD symptoms. Psychoneuroendocrinology.

[3] Garfin DR, Thompson RR, Holman EA (2018). Acute stress and subsequent health outcomes: A systematic review. J. Psychosom. Res..

[4] Ginzburg K, Solomon Z, Koifman B, Keren G, Roth A, Kriwisky M, Kutz I, David D, Bleich A (2003). Trajectories of posttraumatic stress disorder following myocardial infarction: A prospective study. J. Clin. Psychiatry..

[5] Jacquet-Smailovic M, Tarquinio C, Alla F, Denis I, Kirche A, Tarquinio C, Brennstuhl MJ (2021). Posttraumatic stress disorder following myocardial infarction: A systematic review. J. Trauma Stress..

[6] Edmondson D, Rieckmann N, Shaffer JA, Schwartz JE, Burg MM, Davidson KW, Clemow L, Shimbo D, Kronish IM (2011). Posttraumatic stress due to an acute coronary syndrome increases risk of 42-month major adverse cardiac events and all-cause mortality. J. Psychiatr. Res..

[7] Shahrour G, Dardas LA (2020). Acute stress disorder, coping self-efficacy and subsequent psychological distress among nurses amid COVID-19. J. Nurs. Manag..

[8] Cooke BD, Rossmann MM, McCubbin HI, Patterson JM (1988). Examining the definition and assessment of social support: A resource for individuals and families. Fam. Relat..

[9] Norris FH, Kaniasty K (1996). Received and perceived social support in times of stress: A test of the social support deterioration deterrence model. J. Pers. Soc. Psychol..

[10] Park KH, Kim DH, Kim SK, Yi YH, Jeong JH, Chae J, Hwang J, Roh H (2015). The relationships between empathy, stress, and social support among medical students. Int. J. Med. Educ..

[11] Ozer EJ, Best SR, Lipsey TL, Weiss DS (2003). Predictors of posttraumatic stress disorder and symptoms in adults: A meta-analysis. Psychol. Bull..

[12] McMahon G, Creaven AM, Gallagher S (2020). Perceived social support mediates the association between attachment and cardiovascular reactivity in young adults. Psychophysiology.

[13] Bryant RA, Friedman MJ, Spiegel D, Ursano R, Strain J (2011). A review of acute stress disorder in DSM-5. Depress Anxiety..

[14] Cardeña E, Koopman C, Classen C, Waelde LC, Spiegel D (2000). Psychometric properties of the Stanford Acute Stress Reaction Questionnaire (SASRQ): A valid and reliable measure of acute stress. J. Trauma Stress..

[15] Renzi A, Di Trani M, Solano L, Minutolo E, Tambelli R (2020). Success of assisted reproductive technology treatment and couple relationship: A pilot study on the role of romantic attachment. Health Psychol. Open..

[16] Xiao SY (1994). Theoretical basis and research application of “Social Support Rating Scale”. J. Clin. Psychiatry.

[17] Liu JW, Li FY, Lian YL (2008). Reliability and validity of Social Support Rating Scale. J Xinjiang Med. Univ..

[18] von Känel R, Hari R, Schmid JP, Wiedemar L, Guler E, Barth J, Saner H, Schnyder U, Begré S (2011). Non-fatal cardiovascular outcome in patients with posttraumatic stress symptoms caused by myocardial infarction. J. Cardiol..

[19] Sochman J, Fabián J, Málek I, Belán A, Englis M (1989). Creatine kinase kinetics and myocardial infarction in different regions of the left ventricle. Cor. Vasa..

[20] Ryan W, Karliner JS, Gilpin EA, Covell JW, DeLuca M, Ross J (1981). The creatine kinase curve area and peak creatine kinase after acute myocardial infarction: Usefulness and limitations. Am. Heart J..

[21] Whitehead DL, Perkins-Porras L, Strike PC, Steptoe A (2006). Post-traumatic stress disorder in patients with cardiac disease: Predicting vulnerability from emotional responses during admission for acute coronary syndromes. Heart.

[22] Nagata S, Funakosi S, Amae S, Yoshida S, Ambo H, Kudo A, Yokota A, Ueno T, Matsuoka H, Hayashi Y (2008). Posttraumatic stress disorder in mothers of children who have undergone surgery for congenital disease at a pediatric surgery department. J. Pediatr. Surg..

[23] Ren H, Ding Y, Hu H, Gao T, Qin Z, Hu Y, Cao R, Liang L, Li C, Mei S (2020). Relationships among economic stress, social support, age and quality of life in patients with chronic wounds: A moderated mediation model. J. Adv. Nurs..

[24] Ye Z, Yang X, Zeng C, Wang Y, Shen Z, Li X, Lin D (2020). Resilience, social support, and coping as mediators between COVID-19-related stressful experiences and acute stress disorder among college students in China. Appl. Psychol. Health Well Being..

[25] Wang J, Mann F, Lloyd-Evans B, Ma R, Johnson S (2018). Associations between loneliness and perceived social support and outcomes of mental health problems: A systematic review. BMC Psychiatry.

[26] Heenan A, Greenman PS, Tassé V, Zachariades F, Tulloch H (2020). Traumatic stress, attachment style, and health outcomes in cardiac rehabilitation patients. Front. Psychol..

[27] Xiaoyun C, Fenglan L (2020). The relationships among insecure attachment, social support, and psychological experiences in family caregivers of cancer inpatients. Eur. J. Oncol. Nurs..

[28] Herbert J (2013). Cortisol and depression: Three questions for psychiatry. Psychol. Med..

[29] Zerach G, Elklit A (2020). Attachment and social support mediate associations between polyvictimization and psychological distress in early adolescence. Int. J. Psychol..

[30] Girme YU, Overall NC, Simpson JA, Fletcher GJ (2015). "All or nothing": Attachment avoidance and the curvilinear effects of partner support. J. Pers. Soc. Psychol..

[31] Vilchinsky N, Haze-Filderman L, Leibowitz M, Reges O, Khaskia A, Mosseri M (2010). Spousal support and cardiac patients’ distress: The moderating role of attachment orientation. J. Fam. Psychol..

[32] Bryant RA, Friedman MJ, Spiegel D, Ursano R, Strain J (2011). A review of acute stress disorder in DSM-5. Depress. Anxiety..

[33] Roberge MA, Dupuis G, Marchand A (2008). Acute stress disorder after myocardial infarction: Prevalence and associated factors. Psychosom. Med..

